# Modifiable Socio-Behavioural Factors Associated with Overweight and Hypertension among Persons Aged 35 to 60 Years in Eastern Uganda

**DOI:** 10.1371/journal.pone.0047632

**Published:** 2012-10-15

**Authors:** Roy William Mayega, Fredrick Makumbi, Elizeus Rutebemberwa, Stefan Peterson, Claes-Göran Östenson, Göran Tomson, David Guwatudde

**Affiliations:** 1 Department of Public Health Sciences, Division of Global Health (IHCAR), Karolinska Institutet, Stockholm, Sweden; 2 Department of Epidemiology and Biostatistics, Makerere University School of Public Health, Kampala, Uganda; 3 Department of Health Policy, Planning and Management, Makerere University School of Public Health, Kampala, Uganda; 4 Department of Molecular Medicine and Surgery, Endocrine and Diabetes Unit, Karolinska Institutet, Stockholm, Sweden; 5 International Maternal and Child Health Unit, Uppsala University, Uppsala, Sweden; 6 Department of Learning, Informatics, Management and Ethics (LIME), Division of Medical Management Centre (MMC), Karolinska Institutet, Stockholm, Sweden; 7 Iganga-Mayuge Health and Demographic Surveillance Site, Iganga, Uganda; John Hopkins Bloomberg School of Public Health, United States of America

## Abstract

**Background:**

Few studies have examined the behavioural correlates of non-communicable, chronic disease risk in low-income countries. The objective of this study was to identify socio-behavioural characteristics associated with being overweight or being hypertensive in a low-income setting, so as to highlight possible interventions and target groups.

**Methods:**

A population based survey was conducted in a Health and Demographic Surveillance Site (HDSS) in eastern Uganda. 1656 individuals aged 35 to 60 years had their Body Mass Index (BMI) and blood pressure (BP) assessed. Seven lifestyle factors were also assessed, using a validated questionnaire. Logistic regression was used to identify socio-behavioural factors associated with being overweight or being hypertensive.

**Results:**

Prevalence of overweight was found to be 18% (25.2% of women; 9.7% of men; p<0.001) while prevalence of obesity was 5.3% (8.3% of women; 2.2% of men). The prevalence of hypertension was 20.5%. Factors associated with being overweight included being female (OR 3.7; 95% CI 2.69–5.08), peri-urban residence (OR 2.5; 95% CI 1.46–3.01), higher socio-economic status (OR 4.1; 95% CI 2.40–6.98), and increasing age (OR 1.8; 95% CI 1.12–2.79). Those who met the recommended minimum physical activity level, and those with moderate dietary diversity were less likely to be overweight (OR 0.5; 95% CI 0.35–0.65 and OR 0.7; 95% CI 0.49–3.01). Factors associated with being hypertensive included peri-urban residence (OR 2.4; 95%CI 1.60–3.66), increasing age (OR 4.5; 95% CI 2.94–6.96) and being over-weight (OR 2.8; 95% CI 1.98–3.98). Overweight persons in rural areas were significantly more likely to be hypertensive than those in peri-urban areas (p = 0.013).

**Conclusions:**

Being overweight in low-income settings is associated with sex, physical activity and dietary diversity and being hypertensive is associated with being overweight; these factors are modifiable. There is need for context-specific health education addressing disparities in lifestyles at community levels in rural Africa.

## Introduction

Non-communicable diseases (NCDs), such as cardiovascular disease (CVD), are the leading causes of adult mortality globally [1], [2]. The increasing incidence of chronic diseases in low-income countries of sub-Sarahan Africa (SSA) poses a growing challenge to their national health systems [3], given that infectious diseases are still highly prevalent in these settings. The increase is attributed to interrelated changes in demographic and socio-economic determinants, influenced by globalization [4], [5]. Propelling the upsurge of CVD in Africa is the growing prevalence of risk factors, including obesity and hypertension among others [6], [7]. In some countries (e.g. Ghana, South Africa and Cameroon) CVD risk factors have increased to epidemic proportions [8], [9], [10]. In the poorest sub-Saharan Africa countries, the prevalence of overweight has tripled to 10–25% of their populations over the last two decades [7]. Eighty percent of CVD deaths take place in low and middle income countries [11], [12].

Proximate risk factors for CVD, including high blood pressure and being overweight, are largely driven by behavioural factors [13]. However, data on behavioural factors associated with being overweight or being hypertensive in low income countries, including sub-Saharan Africa, are limited [3]. By 2010, only nine countries in Africa (excluding Uganda) had conducted national surveys on NCDs and their risk factors [14]. Most of these surveys do not present an analysis of the link between socio-behavioural factors and being overweight or hypertensive [15]. In three population-based studies on the prevalence of CVD risk factors conducted in Uganda, analysis focused on identifying demographic characteristics associated with the CVD risk factors [16], [17], [18]. Similarly, a study in Cameroon focused only on the association between over-weight and socio-economic status [9]. The lack of a holistic assessment of socio-behavioural factors associated with CVD risk is noticeable in other studies conducted across sub-Sahara Africa [3], [6], [14], [19].

Most of the socio-behavioural characteristics of individuals are modifiable. It is thus important to identify these, to inform policy formulation for CVD risk reduction. The objective of this study was to identify socio-behavioural characteristics associated with being overweight or being hypertensive among people aged 35–60 years.

**Figure 1:**
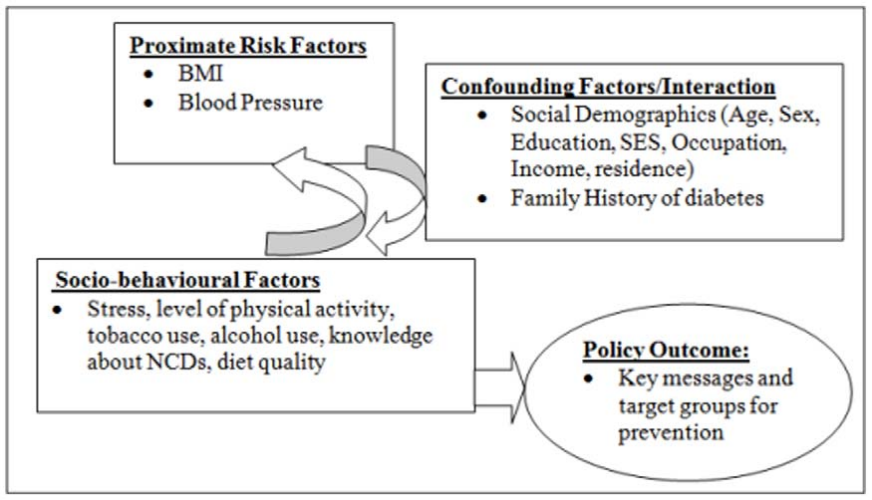
Conceptual framework on socio-behavioural factors likely associated with BMI and blood pressure (Developed by the authors).

## Methods

### Setting

The study was conducted in the Iganga-Mayuge Health and Demographic Surveillance Site (HDSS) [20], located in two eastern Uganda districts of Iganga and Mayuge, approximately 120 kilometres east of Kampala, the capital city of Uganda. The HDSS has a population of approximately 70,000 people in 65 villages. The HDSS has one town comprised of 13 villages (hereafter referred to as ‘peri-urban areas’); the rest of the villages are rural. Data is collected every six months to update the HDSS database. In addition to socio-demographic information, routine data is collected on births, deaths, and in- and out- migrations. Apart from the routine surveillance activities, add-on studies are often conducted within the HDSS, including this study. Data for this study were collected over a 6 week period in May and June 2011.

**Table 1:** Background characteristics of participants.

Characteristic	-n-	%
Sex:		
Males	805	48.6%
Females	851	51.4%
Location of residence:		
Rural	1352	84.1%
Peri-urban	264	15.9%
Age-group:		
35–39	506	30.6%
40–44	397	24.0%
45–49	356	21.5%
50–54	223	13.5%
55–60	172	10.4%
Main occupation:		
Subsistence/Domestic	1010	61.0%
Petty trade	169	10.2%
Commercial agriculture	143	8.6%
Formal salaried	107	6.5%
Casual labour/barter	96	5.8%
Mason/Artisan	45	2.7%
Medium or large trade	19	1.1%
Not specified	67	4.0%
Highest level of education:		
None	322	19.4%
Lower Primary	347	21.0%
Higher Primary	588	35.5%
Secondary – O level	287	17.3%
Secondary – A level	27	1.6%
Tertiary	85	5.1%
Family history of diabetes:		
No	1450	87.6%
Yes	206	12.4%
SES quintiles:		
Lowest	331	20.0%
Second	331	20.0%
Middle	322	19.4%
Fourth	319	19.3%
Highest	353	31.3%

### Study Population

The study population comprised adult men and women aged 35–60 years within the HDSS. A clustered study design [21] was used, villages being the clusters. A sample of 42 villages (8 peri-urban and 34 rural) were selected, from which 1,680 participants were selected proportionate to the village population sizes. From the HDSS database, participants from each village were selected using simple random sampling. HDSS locator information was used to trace participants to their households, by Village Scouts who routinely register vital events. Data were collected by eleven teams, each comprising of two research assistants (a nurse and a social worker). The research assistants underwent three days of rigorous training regarding the objectives of this study, and on administration of the study tools to participants.

### Measurements

Research assistants obtained physical measurements and administered a structured questionnaire. Height was measured (to two decimal places) using standard height meters, with the participant standing upright. Weight was measured using calibrated Seca® scales, with the participant lightly clothed. Waist and hip circumference were measured using a tape measure. Waist circumference was measured around a horizontal plane through the mid-point between the lower costal margin and the iliac crest, waistline unclothed. Hip circumference was measured around a horizontal plane through the trochanters. BMI was calculated as weight in kilograms divided by the square of height in metres. A participant was classified as overweight if their BMI was 25 Kgm^−2^ or greater. A participant was classified as being obese if their BMI was 30 or greater [22]. Further, the waist circumference was also used to classify abdominal obesity. Participants were classified as having a normal waist circumference if it was ≤94 centimetres (cm) for males and ≤80 cm for females. They were classified as having a moderately elevated waist circumference if it was 94.1–102 cm for males and 80.1–88 cm for females, and as having a substantially elevated waist circumference if it was >102 cm for males and >88 cm for females [22].

**Table 2:** Prevalence of overweight and hypertension, by sex.

Characteristic	Males		Females		Overall	p-value
	-n-	%	-n-	%	%	
BMI categories:						
Less than 18.5	142	17.6%	124	14.6%	16.1%	Ref
18.5–24.99	585	72.7%	513	60.3%	66.3%	0.975
25–29.99	60	7.5%	144	16.9%	12.3%	<0.001
30+	18	2.2%	70	8.2%	5.3%	<0.001
Waist circumference:						
Normal	755	93.8%	455	53.5%	73.0%	Ref
Moderately elevated	39	4.8%	179	21.0%	13.2%	<0.001
Substantially elevated	11	1.4%	217	24.5%	13.8%	<0.001
Blood pressure:						
Normal or low	214	26.6%	339	39.8%	33.4%	Ref
Pre-hypertensive	424	52.7%	339	39.8%	46.1%	<0.001
Hypertensive or on treatment	167	20.7%	173	20.4%	20.5%	0.356
Both Hypertensive and Overweight:						
No	772	95.9%	783	92.0%	93.9%	Ref
Yes	33	4.1%	68	8.0%	6.1%	0.001

Ref = Reference category.

Two blood pressure (BP) measurements were taken (at least 5 minutes apart), with the participant seated, using a calibrated electronic BP device (Welch-Allyn®). For each participant's two BP measurements, the average was calculated to represent their BP. A participant was classified as being hypertensive if their average systolic BP was 140mmHg or higher, or if their average diastolic BP was 90mmHg or higher, or if they were on anti-hypertensive treatment [23]. Further, pre-hypertension was defined as having an average systolic BP between 120–139 mmHg, or an average diastolic BP between 80–89 mmHg.

A structured questionnaire was used to collect data on socio-behavioural factors, including demographic characteristics, ownership of 11 indicator assets for purposes of assessing socio-economic status (based on the Uganda Demographic and Health Survey) [24], family history of diabetes, psychosocial stress, physical activity, tobacco and alcohol use, knowledge about lifestyle-diseases and food frequency. Most of the questions were adapted from the WHO Stepwise Approach to NCD Surveillance (STEPS) tool [25]. The WHO STEPS tool has been used for risk factor surveillance in a number of countries in sub-Saharan Africa [26]. The questionnaire was translated into the local language and was pre-tested in a non-study village.

The questionnaire used to assess socio-behavioural characteristics was adapted from different tools that have been validated elsewhere. Questions on physical activity were derived from the STEPs tool, which adapts them from the WHO Global Physical Activity Questionnaire (GPAQ). The GPAQ has been shown to have a high reliability (kappa of 0.67 to 0.73 and 0.84 to 0.93), but a fairly low criterion validity (up to 0.35) [27], [28]. The GPAQ has also been evaluated in Africa. For example, a recent assessment in Nigeria showed that it had good concurrent validity (correlation of 0.78) and reliability (coefficient of 0.73) for vigorous-intensity physical activity, but validity was lower for moderate-intensity activities [29]. Our own evaluation of the items used in the physical activity assessment showed a moderate degree of internal consistency (Cronbach's Alpha = 67%). With regard to the questions on alcohol use and smoking, studies by the WHO have assessed these items to have a moderate to high degree of internal consistency for assessing dependency (correlation coefficients of 0.7–0.9), but reliability is lower when assessing abuse [30], [31]. The questions used to assess psychosocial stress were shown to have an internal correlation of 0.8, but the authors do not provide data on criterion validity [32]. With regard to the validity of the items used to measure knowledge about life-style diseases, our own assessment shows that the three domains used (knowledge of diabetes symptoms, risk factors and ways of prevention) showed excellent internal consistency (Cronbach's Alpha = 82%). The individual dietary diversity score that was used in this study has been demonstrated to have a high level of validity in assessing food quality (coefficients range from 0.33 to 0.97 in various studies) and some of these assessments have been conducted in rural Africa [33], [34].

To classify socio-economic status (SES), principal components analysis (PCA) was run on the 11 household assets evaluated. These assets included owning: 1) a radio, 2) a television, 3) a mobile phone, 4) a bicycle, 5) a motorcycle, 6) a motor vehicle, 7) a piece of land, 8) large farm animals like cattle, goats and sheep, 9) small farm animals like poultry, 10) a manufactured bed, and 11) the nature of the walls of their house. The principal component on which most assets loaded was used to generate an SES score for each participant. Participants were then grouped into SES quintiles (five descending groups). The same approach is used in Demographic Surveillance Surveys in Uganda [24].

The questions on physical activity sought information on the participants' undertaking of ‘vigorous-intensity activities’ (e.g. lifting heavy loads, digging, construction work, etc) and ‘moderate-intensity activities’ (e.g. brisk walking, carrying light loads, riding a bicycle and recreational activities like physical exercises and walking during leisure, etc). Time spent on these activities in a typical week was recorded. Participants were classified into those that met the WHO minimum recommendations for physical activity (at least 75 minutes of vigorous-intensity, or at least 150 minutes of moderate-intensity activities per week) [35].

Psychosocial stress was measured in five domains, including anxiety, apathy, depression, fatigue and insomnia, using 5 questions adapted from Erikson et al [32]. The maximum possible score on this scale was 20. Based on their total score, participants were classified into 3 stress levels by dividing the range of the scale (0–20) into 3 equal parts (0–6 = low stress, 7–13 = Moderate stress and 14–20 = High level of stress).

Knowledge about lifestyle diseases was measured in five domains using diabetes as a proxy. The domains included awareness about diabetes, and its symptoms, its risk factors, its prevention and misconceptions about diabetes. Participants were scored with a “1” for each knowledge item they knew, and with a “0” for those they did not know. The total score for the 39 items used in the assessment was used to grade participants into four knowledge levels, i.e. very-low (0–9), low (10–19), moderate (20–29) and good (30–39). In this analysis, participants in the ‘moderate’ and ‘good’ category were classified as having adequate knowledge about life-style diseases.

Tobacco use was assessed using questions on current and ever use of tobacco, and associated habits, whereas alcohol use was assessed with questions on frequency, type of alcohol and quantity consumed. Participants were classified as engaging in ‘harmful alcohol taking’ if they exceeded the recommended level for safe alcohol in-take i.e. more than 3 drinks on average every time they drink, or if they undertook binge drinking (i.e. more than 3 drinks on one occasion in the one month preceding the survey) [25], [36]. Information was also collected on family history of diabetes, defined as a history of diabetes in one 1^st^ degree or at least two 2^nd^ degree relatives [37].

Food frequency was assessed using dietary recall of foods that an individual ate in the seven days preceding the survey. Local foods were grouped into 9 food groups as recommended for use in dietary diversity assessments [38]. The 9 food groups included: 1) cereals 2) tubers and plantains 3) pulses, 4) vegetables, 5) fruits, 6) milk and dairy products, 7) meats, offal and poultry, 8) fish, and 9) oils/fat. Individual dietary diversity was then assessed by giving a score of “1” for each food group if the participant reported to have eaten at least one food in that group in the week preceding the survey, or a “0” if they did not eat any food in that group [38]. Thus, since we had 9 food groups, each participant could score a maximum food diversity score of 9. For example if a participant reported to have eaten potatoes at least once in the previous week, they would be scored with a “1” under the ‘tubers/plantains’ food group; conversely, they would be scored with a “0” under the ‘tubers/plantains’ food group if they did not eat any food listed in this group. A score of 0–3 was regarded as ‘low dietary diversity’, 4–6 as ‘moderate dietary diversity’ and 7–9 as ‘high dietary diversity’ [38].

### Conceptual framework

Figure 1 lays out the conceptual model that guided our analysis. In the model, we hypothesize that BMI and Blood pressure are associated with socio-behavioural factors. These socio-behavioural factors may be confounded by demographic characteristics. Some of these socio-behavioural factors are modifiable and if identified, could influence policy by clarifying key messages and target groups for education on prevention of overweight and hypertension in low income settings.

### Statistical analysis strategy

Data were double entered in EpiData, cleaned and exported to STATA10 for analysis. The prevalence of over-weight and hypertension were calculated as the percentage over-weight and hypertensive respectively, the total study sample being the denominator. Logistic regression analysis was used to identify the socio-behavioural factors associated with being over-weight (Refer to Figure 1 for the conceptual framework that guided the analysis). Because the distribution of overweight based on waist circumference showed wide disparity between sexes, the authors chose to use BMI for assessing associations. Only factors that were significantly associated with being overweight at bi-variable analysis (p<0.05) were included in the adjusted model. Variables used in analysis were: demographic variables (sex, age, residence location, SES), family history of diabetes, blood pressure level, level of physical activity, knowledge about lifestyle diseases, tobacco use, alcohol use, and dietary diversity. Similar analysis was conducted to identify socio-behavioural factors associated with being hypertensive. We report crude odds ratios (COR), adjusted odds ratios (AOR), and their respective 95% confidence intervals (CI) and p-values, as the measure of association. The goodness-of-fit of the logistic regression models was evaluated using the Hosmer-Lemeshow test [39].

### Ethics statement

The conduct of this study was approved by Makerere University School of Public Health Higher Degrees Research and Ethics Committee, the Swedish Regional Ethics Board (Stockholm) and the Uganda National Council of Science and Technology. Permission was also obtained from the Iganga-Mayuge Health and Demographic Surveillance Site (HDSS) management, and signed full informed consent sought from each participant.

## Results

### Characteristics of participants

Of the 1,680 eligible HDSS residents sampled, 1,656 participated in the study (98.6% response rate), of which 851 (51%) were females. Table 1 shows the distribution of background characteristics of the participants. The mean age of participants was 44 years (Standard Deviation (SD) of 7 years; Inter-quartile range (IQR)  = 38–49 years). Sixty-one percent were involved in subsistence work as the main source of livelihood. Nineteen percent of participants had received no formal education. A family history of diabetes was reported in 12.4% of participants (Table 1). Findings not included the following: A significantly higher proportion of men than women were engaged in occupations other than subsistence work (56% vs. 23% respectively, p-value<0.001). Of the participants with no formal education, 73% were women. Although women were more likely to report a positive family history for diabetes than men, the difference was not statistically significant (p = 0.131).

### Prevalence of overweight and hypertension

Table 2 shows the distribution of overweight and hypertension among participants. The mean BMI of the participants was 22 (SD = 4.1; IQR  = 19.3–23.8). Eighteen percent of participants were overweight, with a marked disparity between sexes (25.2% of women vs. 9.7% of men, p<0.001). Additionally, 5.3% were obese (8.3% of women vs. 2.2% of men, p<0.001) (Table 2). Prevalence of abdominal obesity (using waist-circumference) was 27%, but with a much wider disparity between sexes (47% in women vs. 6% in men, p<0.001). The prevalence of hypertension was 20.5%, with no difference between sexes (OR 1.0; 95% CI 0.77–1.24). Forty-six percent of participants were found to be pre-hypertensive (Table 2).

### Factors associated with being overweight

Table 3 shows the distribution of background and socio-behavioural factors associated with being overweight. The non-modifiable factors found to be associated with being overweight included sex, age, and family history of diabetes. Women were about 4 times more likely to be overweight compared to men (OR 3.7; 95% CI 2.69–5.08). Compared to participants aged 35–39, those aged 45–49 and 50–54 years were more likely to be overweight (OR 1.6; 95% CI 1.07–2.40; OR 1.8; 95% CI 1.12–2.79 respectively). Participants with a family history of diabetes were 1.8 times more likely than those without a family history of diabetes, to be overweight (OR 1.8; 95% CI 1.28–2.54).

**Table 3:** Factors associated with being overweight.

Factors	Sub-category	-n-	% Over-weight	COR[95% CI]	AOR†[95% CI]	p-value
**Background**						
Sex:	Male	805	9.7%	1.0	1.0	
	Female	848	25.2%	3.1[2.36–4.19]	3.7[2.69–5.08]	<0.001
Residence:	Rural	1390	14.2%	1.0	1.0	
	Peri-urban	263	35.7%	3.3[2.48–4.52]	2.1[1.46–3.01]	<0.001
Age-group:	35–39	505	14.5%	1.0	1.0	
	40–44	396	18.2%	1.3[0.92–1.89]	1.2[0.82–1.79]	0.338
	45–49	355	18.9%	1.4[0.96–1.98]	1.6[1.07–2.40]	0.023
	50–54	223	21.5%	1.6[1.08–2.43]	1.8[1.12–2.79]	0.015
	55–60	172	18.6%	1.4[0.86–2.14]	1.6[0.92–2.62]	0.093
SES quintile:	Lowest	331	7.7%	1.0	1.0	
	Second	331	17.1%	2.5[1.50–4.12]	2.5[1.50–4.31]	0.001
	Middle	322	20.8%	3.2[1.91–5.19]	3.4[2.03–5.85]	<0.001
	Fourth	319	16.9%	2.4[1.47–4.07]	2.4[1.36–4.08]	0.002
	Highest	353	24.7%	4.0[2.42–6.44]	4.1[2.40–6.98]	<0.001
Family history of diabetes:	No	1447	16.5%	1.0	1.0	
	Yes	206	26.2%	1.8[1.28–2.54]	1.5[1.02–2.22]	0.040
Hypertensive:	No	1314	14.5%	1.0	1.0	
	Yes	339	29.8%	2.5[1.88–3.31]	2.5[1.79–3.38]	<0.001
**Behavioural**						
Attains WHO minimum physical activity level:	No	261	28.0%	1.0	1.0	
	Yes	1392	15.7%	0.5[0.35–0.65]	0.7[0.47–0.99]	0.048
Stress level:	Low	715	16.8%	1.0		
	Moderate	657	19.0%	1.2[0.88–1.53]		
	High	263	16.7%	1.0[0.68–1.45]		
Knowledge about lifestyle diseases:	Very Low	371	11.3%	1.0	1.0	
	Low	715	19.4%	1.9[1.30–2.74]	1.7[1.13–2.55]	0.010
	Moderate	468	18.4%	1.8[1.19–2.62]	1.5[0.95–2.31]	0.086
	Good	99	25.3%	2.6[1.52–4.61]	2.0[1.03–3.70]	0.040
Tobacco user:	No	1554	18.5%	1.0	1.0	
	Yes	99	4.0%	0.2[0.07–0.51]	0.4[0.13–1.11]	0.077
Harmful alcohol taker:	No	1570	18.0%	1.0	1.0	
	Yes	83	10.8%	0.6[0.27–1.12]	0.7[0.33–1.56]	0.399
Dietary diversity:	Low	350	21.1%	1.0	1.0	
	Moderate	1138	16.7%	0.7[0.55–1.01]	0.7[0.49–0.97]	0.033
	High	165	17.0%	0.8[0.47–1.23]	0.8[0.46–1.34]	0.369

Hosmer-Lemeshow Goodness-of-Fit p-value = 0.128; COR = Crude Odds Ratio; AOR = Adjusted Odds Ratio; **†** adjusted for sex, age, residence, SES quintile, family history of diabetes, blood pressure level, level of physical activity, knowledge about lifestyle diseases, tobacco use, alcohol use, and dietary diversity.

Modifiable factors found to be associated with being overweight included location of residence, socio-economic status, blood pressure, level of physical activity, knowledge about diabetes, and dietary diversity. Peri-urban residents were 2 times more likely to be overweight compared to rural residents (OR 2.1; 95% CI 1.46–3.01). The likelihood of being overweight increased significantly with socio-economic status. Participants in the highest SES quintile were 4 times more likely to be overweight than those in the lowest quintile (OR 4.1; 95% CI 2.40–6.98). Of the 1656 participants, 1392 (84%) met the WHO minimum recommendations for physical activity, with no significant difference between sexes (OR 1.2; 95% CI 0.92–1.57) <this finding is not included in the tables>. Participants who met the WHO minimum standard for physical activity were significantly less likely to be overweight than those who did not meet the minimum recommendation (OR 0.7; 95% CI 0.47–0.99). Participants with moderate and higher dietary diversity were also less likely to be overweight than those with low dietary diversity. As a paradoxical finding, people who knew more about diabetes were more likely to be overweight compared to the less knowledgeable.

Factors not significantly associated with being over-weight included occupation, level of education <Finding not included in the tables>, stress level, tobacco use and harmful alcohol intake.

### Factors associated with being hypertensive

Table 4 shows the distribution of background and socio-behavioural factors associated with being hypertensive. Age was the only non-modifiable factor found to be associated with being hypertensive, the level of association increased with age. Compared with the age-group 35–39 years, statistically significant differences in the likelihood of hypertension were noted from the age-group 45–49 years and above. Participants in the age-group 55–60 years were about 5 times more likely to be hypertensive than those in the age-group 35–39 (OR 4.5; 95% CI 2.94–6.96). Socio-economic status, occupation, level of education <not shown in the table> and family history of diabetes were not significantly associated with being hypertensive (Table 4).

**Table 4:** Factors associated with being hypertensive.

Factors	Sub-category	-n-	% Hypertensive	COR[95% CI]	AOR‡[95% CI]	p-value
**Background**						
Sex:	Male	805	20.7%	1.0		
	Female	848	20.3%	1.0[0.77–1.24]		
Residence:	Rural	1390	19.0%	1.0	1.0	
	Peri-urban	263	28.5%	1.7[1.25–2.28]	2.4[1.60–3.66]	<0.001
Age-group:	35–39	505	11.3%	1.0	1.0	
	40–44	396	16.9%	1.6[1.09–2.34]	1.4[0.97–2.12]	0.068
	45–49	355	25.3%	2.7[1.83–3.86]	2.5[1.73–3.69]	<0.001
	50–54	223	28.7%	3.2[2.10–4.78]	3.0[1.97–4.49]	<0.001
	55–60	172	36.0%	4.4[2.88–6.85]	4.5[2.94–6.96]	<0.001
SES quintile:	Lowest	331	19.0%	1.0		
	Second	331	19.6%	1.0[0.70–1.53]		
	Middle	322	22.0%	1.2[0.82–1.78]		
	Fourth	319	22.5%	1.2[0.85–1.82]		
	Highest	353	18.6%	0.9[0.66–1.44]		
Family history of diabetes:	No	1450	20.1%	1.0		
	Yes	206	23.8%	1.2[0.88–1.76]		
BMI:	<25	1361	17.5%	1.0		
	≥25	292	34.6%	2.5[1.89–3.30]	2.8[1.98–3.98]	<0.001
**Behavioural**						
Attains WHO minimum physical activity level:	No	261	22.6%	1.0	1.0	
	Yes	1395	20.1%	0.9[0.63–1.19]	1.2[0.81–1.67]	0.401
Stress level:	Low	717	20.1%	1.0		
	Moderate	676	19.5%	1.0[0.74–1.26]		
	High	263	24.3%	1.3[0.91–1.79]		
Knowledge about lifestyle diseases:	Very Low	371	16.1%	1.0	1.0	
	Low	715	19.4%	1.3[0.90–1.75]	1.1[0.81–1.62]	0.453
	Moderate	468	22.0%	1.5[1.03–2.08]	1.3[0.88–1.85]	0.204
	Good	99	30.3%	3.3[1.96–5.36]	2.7[1.63–4.63]	<0.001
Tobacco user:	No	1554	20.4%	1.0	1.0	
	Yes	99	22.2%	1.1[0.68–1.82]	1.3[0.80–2.28]	0.267
Harmful alcohol taker:	No	1570	20.0%	1.0	1.0	
	Yes	83	24.1%	1.3[0.92–1.77]	0.9[0.52–1.64]	0.779
Dietary diversity:	Low	350	18.6%	1.0	1.0	
	Moderate	1141	21.9%	1.2[0.91–1.67]	1.4[0.98–1.89]	0.058
	High	165	15.2%	0.8[0.47–1.30]	0.8[0.50–1.44]	0.534

Goodness-of-Fit p-value = 0.863; **‡** COR = Crude Odds Ratio; AOR = Adjusted Odds Ratio; adjusted for age, residence, BMI, level of physical activity, knowledge about lifestyle diseases, tobacco use, alcohol use, and dietary diversity.

The modifiable factors found to be associated with being hypertensive included location of residence and being overweight (Table 4). Except for knowledge about life-style diseases, none of the socio-behavioural characteristics (physical activity, level of stress, tobacco use, excessive alcohol intake or dietary diversity) were associated with being hypertensive.

As with BMI, participants who knew more about lifestyle diseases were more likely to be hypertensive, compared to the less knowledgeable (Table 4). On exploring this paradox further, it was found that although level of education was not independently associated with BMI level and hypertension, there was a statistically significant relationship between level of education and level of knowledge about life-style diseases. Participants who were educated above the level of primary were significantly more likely to be in the higher knowledge category that in the lower categories (p<0.001) <not shown in the tables>.

Stratified analysis shows that not only was there an association between being hypertensive and being overweight (OR 2.5, 95% CI 1.89–3.30), but the association was significantly higher among rural residents (OR 2.9, p<0.001) than peri-urban residents (OR 1.3, p = 0.363) (p-value for homogeneity of OR = 0.013). This finding therefore shows that the relationship between being hypertensive and being over-weight is modified by interaction between BMI status and place of residence. Participants who are over-weight are more likely to be hypertensive, but this was observed only in the rural residents and not in the peri-urban areas.

## Discussion

This study not only assesses the prevalence of overweight and hypertension but also describes the modifiable socio-behavioural factors associated with these in a low-income setting. The finding that 18% of participants were overweight shows a high burden of overweight among people aged 35–60 years. Compared with findings from a rural cohort in southern Uganda where 11% of the general population were overweight [17], these findings imply that the likelihood of being overweight may be significantly higher in people aged 35–60 years than in the general population. However, these rates are lower than those reported in rural South Africa [40], and in higher-income countries [41]. Prevalence of hypertension in this population was 21%, consistent with other studies in sub-Saharan Africa [10], [17], [18], [42]. In addition, the finding that 46% of participants were pre-hypertensive demonstrates the relevance of targeted prevention through life-style measures. The high burden of overweight and hypertension suggest an increased risk for non-communicable diseases like type-2 diabetes and CVD, and hence the need for these to be addressed by the health system.

The non-modifiable factors found to be associated with being overweight included sex, age and family history of diabetes. Sex was the most significant of these factors, implying a possible connection with gender related factors. This is consistent with findings from other settings in sub-Saharan Africa (SSA) [9], [17], [43], [44]. In contrast to our findings, estimates for most higher-income countries show that obesity is higher in men [15]. Studies have shown that there are positive socio-cultural attitudes towards being overweight among women in SSA [45], [46]. However, the WHO recommended cut-offs for classifying abdominal obesity may not be appropriate for African women [47]. The finding that increasing age is associated with being overweight may be related to increased sedentariness with age, which is a modifiable factor. Similar findings were demonstrated in a study in Morocco [48]. The importance of these non-modifiable factors lies in defining whom to target for prevention of overweight at primary care levels in low income countries.

This study affirms that age is an important non-modifiable factor associated with hypertension, similar to what was found in other contexts in sub-Saharan Africa [18], [49], [50]. The increased likelihood of hypertension from 45–49 years and older age-groups has important implications for targeted screening. Decisions about the target age-group for primary care level screening for hypertension could be guided by this finding. Similar to a study in South Africa [10], sex was not significantly associated with hypertension. This contrasted with findings from a study in Rukungiri in rural western Uganda [18]. However, the Rukungiri study targeted a wider age-range (18 years and above). Socio-economic status was not found to be associated with hypertension, a finding that has been demonstrated in other studies [18], [44].

The modifiable risk factors associated with being overweight included location of residence, socio-economic status, physical activity and dietary diversity. The observed increase in likelihood of being overweight with socio-economic status and the rural-urban divide may be attributed to ability to afford the more expensive energy-dense foods [51]. Our study population could be in the early phases of the nutritional transition, where obesity is more frequent in wealthier individuals [9], [49], [50], [52]. The link between physical activity and being overweight has been demonstrated in urban settings in sub-Saharan Africa [16], [44]. Our study also shows that 84% of participants meet the minimum recommended physical activity level. STEPS surveys in 22 African countries show that the majority of the population meet the minimum physical activity requirement, but with variability between countries, ranging from 46.8% in Mali to 96.0% in Mozambique [26]. The finding that moderate diversity diets were associated with a lower likelihood of being overweight could mean that diverse diets are more likely to include vegetables and fruits [53]. In urban Benin, dietary quality did not show significant association with obesity. This was attributed to their population being in the early phases of the nutritional transition [44]. Modifiable factors associated with hypertension included location of residence and being overweight. The finding that overweight people in rural areas were significantly more likely to be hypertensive than those in peri-urban areas has implications for targeted screening.

Our study finds that those more knowledgeable about lifestyle diseases were more likely to be overweight and hypertensive. This paradox may be due to hypertensive people knowing more about lifestyle diseases than non-hypertensives. However, it may suggest that knowledge alone may not be sufficient to change behaviour. The finding that harmful alcohol taking and tobacco use were not associated with being overweight or hypertensive could be due to the low volumes used in this population, or that the sample sizes for these sub-populations were not sufficient.

We recognize a number of methodological limitations in this study, including using knowledge about diabetes as a proxy for knowledge about lifestyle diseases, categorisation of variables that were initially measured as numerical variables, and use of BMI to test for associations, despite its known shortfalls. However, BMI categorizations have been calibrated and recommended by the WHO as a measure of CVD risk, and are widely used in NCD risk assessments, including the STEPs. All categorisations (blood pressure, physical activity and BMI) were based on the standard criteria recommended by the WHO. Limitations arising from using self-reports to assess lifestyles are also noted, but were mitigated by using validated tools. In addition, dietary assessment was limited to dietary diversity scores that do not take into account the quantity of nutrients eaten. A full assessment of nutritional factors was outside the scope of this study. The study was conducted in a HDSS setting, where populations know that they are under observation. However, there are no on-going interventions on NCDs.

## Conclusions

This predominantly rural population in a low income setting has a high prevalence of overweight and hypertension. Being overweight in this setting is associated with insufficient physical activity and low dietary diversity while being hypertensive is associated with being overweight; these factors are modifiable. The policy implication of these findings is that primary health care programmes should integrate education on these lifestyle risk factors. Messages should emphasize culturally relevant ways of increasing physical activity and balanced diets. Because women are more likely to be over-weight, interventions should incorporate a gender dimension. The increased likelihood of hypertension in age groups above 45 years and in overweight persons justify the need for routine screening of people older than 45 years for hypertension, especially if they are overweight.

### Key Messages

- About one in six people between 35 and 60 years of age in this low-income setting are overweight; being overweight in low-income settings is associated with gender factors, insufficient physical activity, low dietary diversity, peri-urban residence and socio-economic status, which are modifiable risk factors.
- About one in five people aged 35 and 60 years in this low-income setting is likely hypertensive; hypertension is associated with being overweight, especially among rural dwellers, and being overweight is modifiable too.
- Targeted education that specifies lifestyle measures regarding diet, physical activity and overweight should be instituted at community level in low income countries.
